# Incidence, Predictors and Outcome of Prosthesis-Patient Mismatch after Transcatheter Aortic Valve Replacement: a Systematic Review and Meta-analysis

**DOI:** 10.1038/s41598-017-15396-4

**Published:** 2017-11-08

**Authors:** Yan-biao Liao, Yi-jian Li, Li Jun-li, Zhen-gang Zhao, Xin Wei, Jiay-yu Tsauo, Tian-yuan Xiong, Yuan-ning Xu, Yuan Feng, Mao Chen

**Affiliations:** Department of Cardiology, West China Hospital, Sichuan University, 37 Guoxue Street, Chengdu, 610041 P. R. China

## Abstract

The aim of this study was to investigate the incidence, predictors and outcome of prosthesis-patient mismatch (PPM) following transcatheter aortic valve replacement (TAVR). A total of 30 articles incorporating 4,691 patients were identified. The pooled incidences of overall, moderate and severe PPM following TAVR were 33.0%, 25.0% and 11.0% respectively. Medtronic CoreValve (MCV) had lower incidence of overall (32% vs: 40%, P < 0.0001) and moderate (23% vs 32%, P < 0.0001) than Edwards Sapien (ESV). PPM was associated with a younger age, smaller annulus diameter and lower left ventricular ejection fraction in comparison with those patients without PPM. Post-dilation (OR, 0.51, 95% CI, 0.38 to 0.68, p < 0.001) during TAVR would decrease the incidence of PPM. Although PPM was common after TAVR, no significant differences were observed both in short- and mid-term all-cause mortality (30 day: OR: 1.1, 95% CI, 0.70 to 1.73 and 2 year: OR: 1.01, 95% CI, 0.74 to 1.38) between patients with PPM and those without PPM. In conclusion, despite being common after TAVR, the incidence of PPM was lower than that of surgical aortic valve replacement (SAVR) and decreased with the experience accumulating, and PPM was not seen to impact on short- and mid-term survival, regardless of its magnitude.

## Introduction

Prosthesis-patient mismatch (PPM) is present when the prosthetic valve is too small in relation to body size and was first described by Rahimtoola^1^. The severity of PPM was evaluated by the indexed effective orifice area, PPM was defined if indexed EOA was less than 0.85 cm^2^/m^2^, moderate PPM defined as ≥0.65 cm^2^/m^2^ and ≤0.85 cm^2^/m^2^, and severe PPM defined as <0.65 cm^2^/m^2^ ^1^. Several studies have reported that the prevalence of PPM after surgical aortic valve replacement (SAVR) ranged from 20% to 70%^2^, and the higher pressure gradients observed in PPM^3^ results in reduced reverse remodeling^4^. Besides, two recent meta-analysis reported that moderate and severe PPM after SAVR was associated with higher overall mortality^5,6^.

Transcatheter aortic valve replacement have developed rapidly as an alternative technique to treating patients with severe aortic stenosis. And Increasing evidence demonstrated TAVR have comparable results in patients with intermediate surgical risk, compared to SAVR^7,8^. Thus, the incidence and outcome of PPM after TAVR is concerned when TAVR extended to patients with intermediate surgical risk. However, controversial has intensified on the incidence, predictors and outcome of PPM after TAVR^9–11^. Therefore, we performed a systematic review and meta-analysis to comprehensively and quantitatively investigate the incidence, predictors, preventive approaches and outcome of PPM.

## Results

### Literature search and study selection

**Figure 1:**
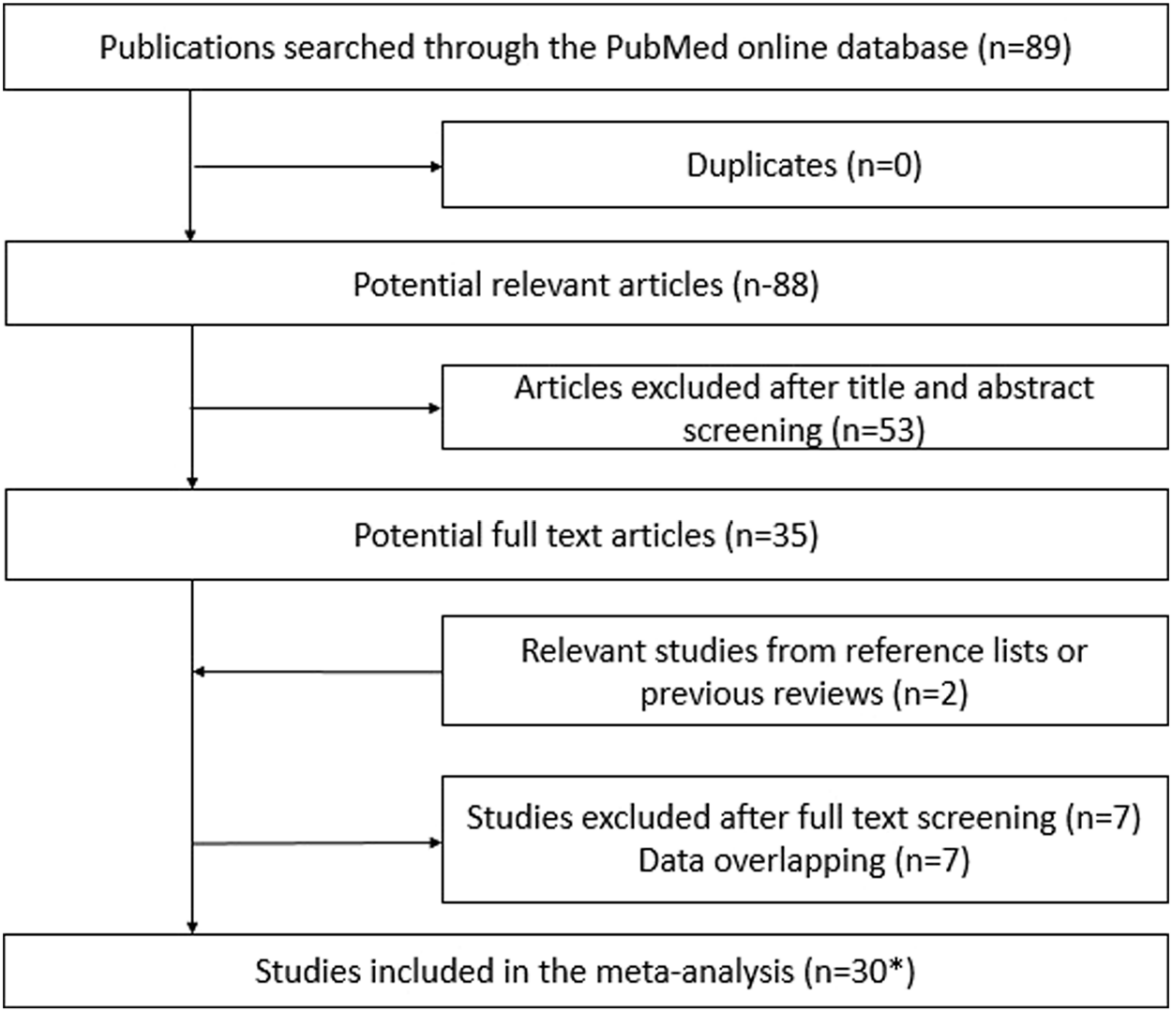
Flow diagram of citation research and selection. *Indicate that one study reporting two clusters of patients including randomized and non-randomized clinical trial which was regarded as two independent studies.

The process of study selection was illustrated in Fig. 1. There were 35 studies left after screening the titles and abstracts. After removing overlapping data, a total of 30 studies^9–37^ incorporating 4,691 patients were eligible. There was 1 study^11^ reporting two clusters of patients including randomized and non-randomized clinical trial which was regarded as two independent studies. The characteristics of the included overall studies were shown in Supplemental Table 1.

### Quality assessment

The quality of eligible cohort studies were assessed using the NOS scale, while quality of single-arm studies were evaluated by using Cross-Sectional/Prevalence Study Quality. Overall quality of these eligible studies was good.

### Incidence of PPM and subgroup analysis

The pooled incidence of overall, moderate and severe PPM after TAVR was 33.0%, 25.0% and 11.0% separately (Supplemental Table 2). Compared with Edwards Sapien valve (ESV), Medtronic CoreValve (MCV) was associated with lower occurrence rate of overall (MCV: 32% vs ESV: 40%, P < 0.0001), moderate (23% vs 32%, P < 0.0001) but not severe PPM (10% vs 12%, P = 0.15) (Supplemental Table 2).

Other subgroup analysis were presented in Supplemental Table 2. Generally, a reduced trend in the occurrence rate of overall, moderate and severe PPM was seen in patients who were referred to TAVR later (later vs early, overall: 31% vs 38%, P = 0.001; moderate: 23% vs 26%, P = 0.12; severe: 10% vs 15%, P = 0.0005). Patients with higher risk (Logistic EuroSCORE > 20%) had similar incidence of overall, moderate and severe PPM with patients with lower risk (higher vs lower, overall: 30% vs 35%, P = 0.08; moderate: 24% vs 23%, P = 0.54; severe: 12% vs 10%, P = 0.11). The results of pooled estimate above were stable, albeit significant heterogeneity available in some of them.

### TAVR vs SAVR

**Figure 2:**
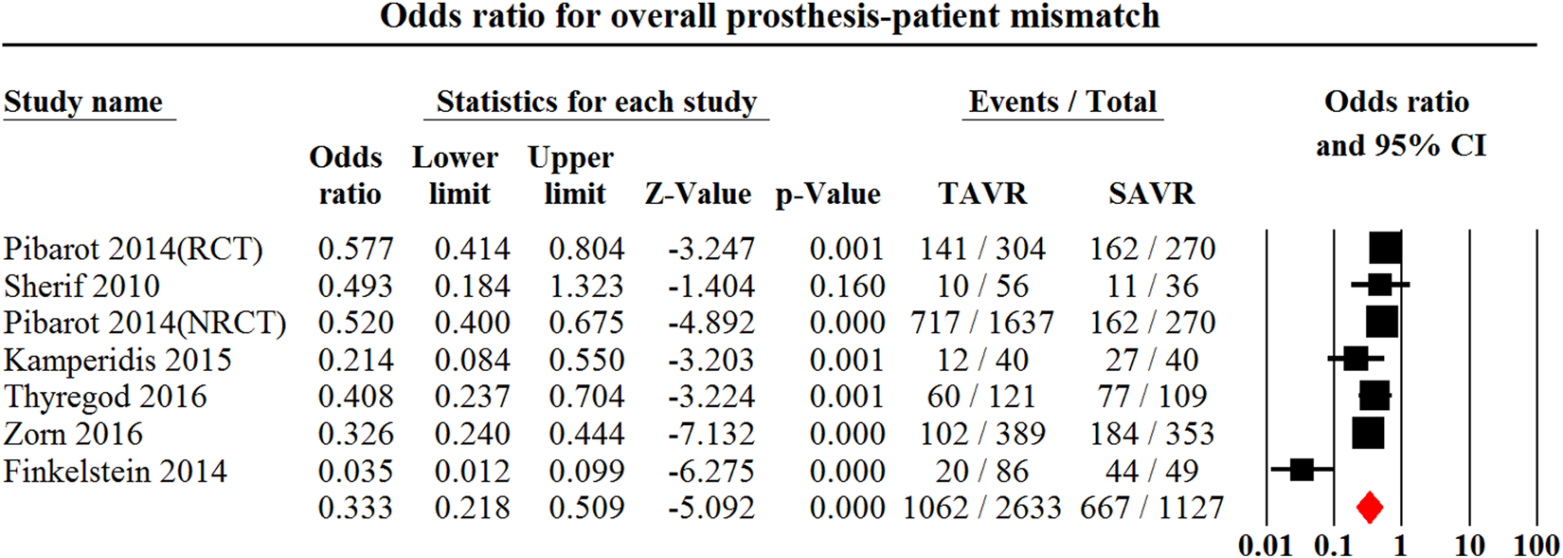
Odds ratio for overall Prosthesis-Patient Mismatch Comparing Transcatheter Aortic Valve replacement with Surgical Aortic Valve Replacement. PPM indicates Prosthesis-Patient Mismatch; TAVR indicates Transcatheter Aortic Valve Replacement.

**Figure 3:**
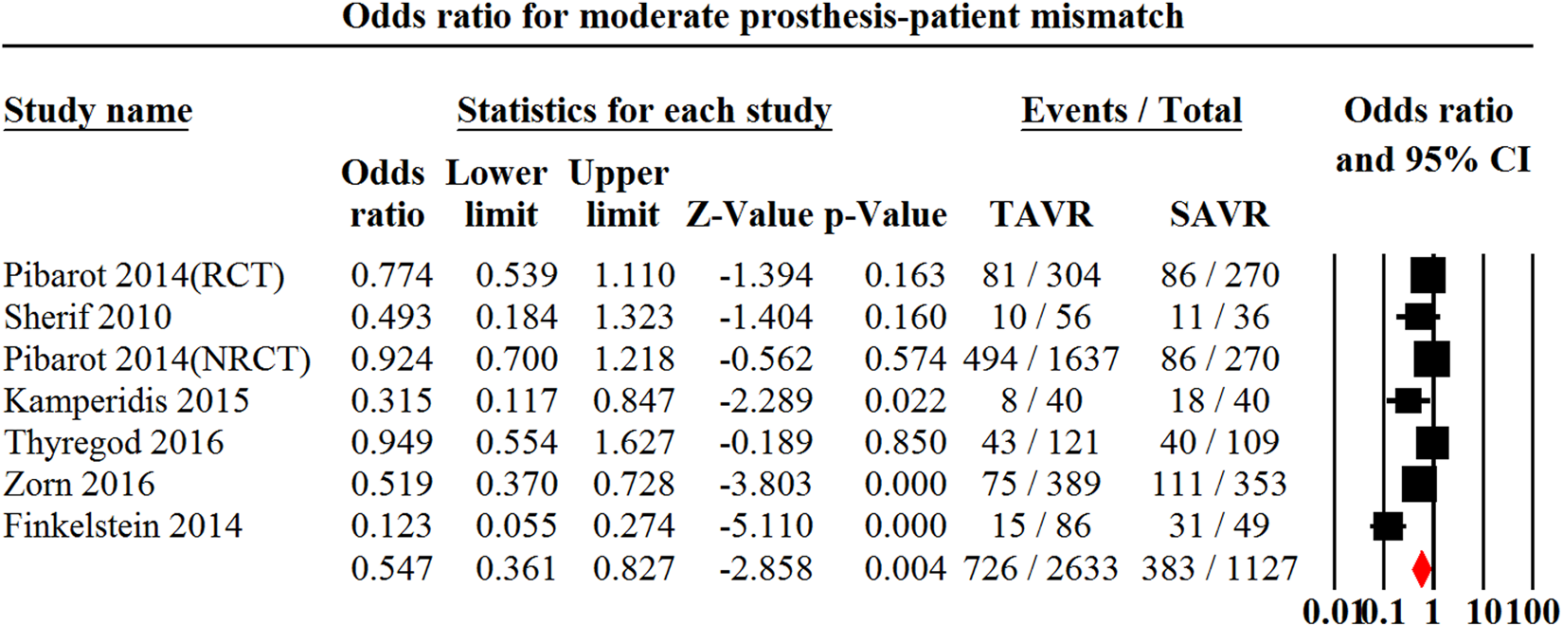
Odds ratio for moderate Prosthesis-Patient Mismatch Comparing Transcatheter Aortic Valve replacement with Surgical Aortic Valve Replacement. PPM indicates Prosthesis-Patient Mismatch; TAVR indicates Transcatheter Aortic Valve Replacement.

**Figure 4:**
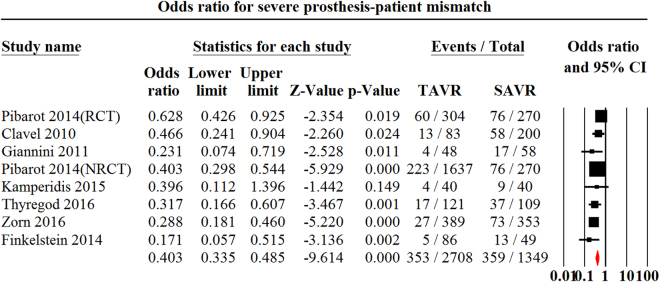
Odds ratio for severe Prosthesis-Patient Mismatch Comparing Transcatheter Aortic Valve replacement with Surgical Aortic Valve Replacement. PPM indicates Prosthesis-Patient Mismatch; TAVR indicates Transcatheter Aortic Valve Replacement.

There were 7^11,13,26,31,33,34^, 7^11,13,26,31,33,34^ and 8^11,21,22,26,31,33,34^ studies recruiting 3,760, 3,760 and 4,057 patients that reported the incidences of overall, moderate and severe PPM after TAVR compared to SAVR respectively. TAVR had lower incidence of overall (OR, 0.33, 95% CI, 0.22 to 0.51, P < 0.001, I^2^ = 81.4, egger’s = 0.09, Fig. 2), moderate (OR, 0.55, 95% CI, 0.36 to 0.83, P = 0.003, I^2^ = 80.0, egger’s = 0.13, Fig. 3) and severe PPM (OR, 0.39, 95% CI, 0.33 to 0.48, P < 0.001, I^2^ = 36.0, egger’s = 0.17, Fig. 4) than SAVR.

### Predictive and preventive factors

In order to investigate the predictors of PPM, we pooled 14^9–12,15,17,19,20,23,28,31–33^ cohort studies incorporating 1,454 patients suffering from PPM and 2,324 patients free from PPM using the univariate analysis method (Table 1). Patients with PPM were younger than those patients without PPM (PPM: 80.8 yrs vs No-PPM: 82.5 yrs, p < 0.001). The PPM group was associated with larger body surface area (BSA) (PPM: 1.85 m^2^ vs No-PPM: 1.74 m^2^, p < 0.001), larger body mass index (BMI) (PPM: 28.1 kg/m^2^ vs No-PPM: 25.9 kg/m^2^, p < 0.001), smaller aortic annulus diameters (PPM: 21.5 mm vs No-PPM: 21.8 mm, p = 0.03), lower baseline left ventricular ejection fraction (LVEF) (52.7 vs 55.7, p < 0.001), smaller baseline EOA (PPM: 0.63 cm^2^ vs No-PPM: 0.68 cm^2^, p = 0.005) and smaller baseline indexed EOA (0.32 cm^2^/m^2^ vs 0.38 cm^2^/m^2^, p < 0.001) in comparison with that of No-PPM group. Other variables were insignificant and presented in Table 1.

**Table 1:** Pooled characteristics of patients with and without Prosthesis-Patient Mismatch.

Characteristics	PPM	No-PPM	Number of studies	Number of patients	P value
Age	80.8 ± 0.8	82.5 ± 0.7	14^9–12,15,17,19,20,23,28,31–33^	3,778	<0.001
Male, n/total (%)	591/1454 (40.6%)	1151/2324 (49.5%)	10^9–12,15,17,19,20,23^	3,778	<0.001
BSA (m^2^)	1.85 ± 0.02	1.74 ± 0.01	10^9–12,15,17,19,20,23^	3,290	<0.001
BMI (Kg/m^2^)	28.1 ± 0.5	25.9 ± 0.4	7^10–12,15,17,20^	3,389	<0.001
Logistic EuroSCORE (%)	21.6 ± 2.2	21.8 ± 2.1	8^9–12,15,20,23^	3,469	0.634
Previous MI, n/total (%)	267/973 (27.4%)	320/1373 (23.3%)	5^11,12,15,20^	2,346	0.29
Aortic annulus diameters(mm)	21.5 ± 0.5	21.8 ± 0.5	9^10–12,15,17,19,20,23^	3,498	0.03
LVEF (%)	52.7 ± 0.8	55.7 ± 0.9	7^11,12,15,19,20,23^	2,586	<0.001
Mean gradient (mmHg)	46.1 ± 1.0	46.3 ± 0.7	8^9,11,12,15,19,20,23^	3,325	0.23
EOA (cm^2^)	0.63 ± 0.02	0.68 ± 0.02	5^9,12,15,19,20^	1,151	0.005
Indexed EOA (cm^2^/m^2^)	0.32 ± 0.01	0.38 ± 0.01	4^12,15,20,23^	1,107	<0.001
Post-balloon dilation	119/345 (34.5%)	724/1553 (46.6%)	3^11,19,32^	1,898	<0.001
Discharge					
LVEF (%)	56.2 ± 0.9	56.8 ± 0.7	3^10,20,23^	772	0.36
Mean gradient (mmHg)	11.3 ± 1.0	8.6 ± 1.0	6^9,10,12,19,20,23^	1,023	<0.001
EOA (cm^2^)	1.25 ± 0.06	1.86 ± 0.06	3^10,12,19^	698	<0.001
Indexed EOA (cm^2^/m^2^)	0.69 ± 0.03	1.09 ± 0.04	4^10,12,19,20^	862	<0.001

Abbreviations: BSA body surface area; MI myocardial infarction; EOA effective orifice area; LVEF left ventricular ejection fraction.

Only optimal implant position^17^ (univariate, p = 0.015) and post-dilation^11,19,32^ (univariate, OR, 0.51, 95% CI, 0.38 to 0.68, I^2^ = 0, p < 0.001) were reported to be associated with reduced incidence of PPM.

### Outcome of PPM

Pooled studies revealed that patients with PPM were associated with higher mean transvalvular gradient, EOA and indexed EOA after TAVR, compared to those patients without PPM (Table 1). Additionally, no significant difference was noted between patients with PPM and those without PPM in both short-term and mid-term all-cause mortality (PPM vs No-PPM: 30 day: OR: 1.17, 95% CI, 0.75 to 1.84, I^2^ = 0; 1 year: OR: 1.14, 95% CI, 0.92 to 1.42, I^2^ = 12.5 and 2 years: OR: 0.98, 95% CI, 0.72 to 1.32, I^2^ = 4.1) (Table 2). The direction of the results above kept consistent when omitting individual studies from the analysis. Furthermore, we found that patients with moderate and severe PPM were not associated with higher risk of 1-year (moderate, OR: 1.0, 95% CI, 0.77 to 1.29, I^2^ = 0; severe, OR: 1.93, 95% CI, 0.76 to 4.91, I^2^ = 81.9) and 2-year all-cause mortality (moderate, OR: 1.05, 95% CI, 0.72 to 1.54, I^2^ = 5; severe, OR: 1.95, 95% CI, 0.38 to 9.91) respectively in comparison with that of PPM (Table 2).

**Table 2:** Pooled impact of Prosthesis-Patient Mismatch on all-cause mortality after transcatheter aortic valve replacement.

Subgroup	No. of studies	No. of patients	ORs	I^2^ for heterogeneity	Model	P for Egger’s
Outcome of overall PPM on al-cause mortality						
30 days	10^9,10,12,15,19,23,28,33^	3,209	1.17 (0.75–1.84)	0	Fixed	0.36
1 year	8^10,11,15,20,23,28,33^	3,125	1.14 (0.92–1.42)	12.5	Fixed	0.37
2 years	6^10,11,15,23,28,31^	1,077	0.98 (0.72–1.32)	4.1	Fixed	0.94
Outcome of moderate PPM on al-cause mortality						
1 year	4^10,11,15^	2,061	1.00 (0.77–1.29)	0	Fixed	0.32
2 years	3^10,11,15^	646	1.05 (0.72–1.54)	5	Fixed	0.43
Outcome of severe PPM on all-cause mortality						
1 year	5^10,11,15,33^	2,041	1.93 (0.76–4.91)	81.9	Random	0.46
2 years	4^10,11,15,31^	653	1.95 (0.38–9.91)	86.6	Random	0.60

Abbreviations: PPM Prosthesis-Patient Mismatch; OR Odds Ratio.

## Discussion

The main results of the present meta-analysis and systematic review were: 1) more than thirty percent of patients underwent TAVR may encounter PPM; 2) MCV had lower prevalence of overall and moderate PPM than ESV; 3) TAVR was associated with reduced risk for the incidence of overall, moderate and severe PPM in comparison with SAVR; 4) baseline larger BMI, smaller aortic annulus diameters, lower LVEF and smaller baseline EOA were associated higher risk for PPM; 5) post-dilation during TAVR was associated with reduced risk for PPM; 6) compared with No-PPM, PPM was not associated with higher short- and mid-term all-cause mortality, regardless of its magnitude.

PPM is a frequent phenomenon after SAVR with the reported incidence ranging from 20% to 70%^2,11^, while, the impact of PPM on patients’ prognosis is still controversial^5,6,38^. Promisingly, TAVR was associated with lower risk in the prevalence of overall, moderate and severe PPM referring to that of SAVR in the present meta-analysis. Additionally, the incidence of PPM was significant lower after TAVR in patients with smaller native aortic annulus (<20 mm)^11,16,39^ but not in patients with larger aortic annulus (≥20 mm)^39^. This difference may be explained by a less frequent occupation of native aortic annulus due to absence of sewing ring^11,40^ which resulted in better hemodynamic results^21,41^. Furthermore, patients underwent SAVR always have more selected size of valve, while patients underwent TAVR have limited size of valve. Besides, oversizing in aortic annulus diameter is commonly recommend in TAVR. These may jointly result in a larger transcatheter valve being inserted in smaller individuals with a small annulus thus may reducing the occurrence of severe PPM^13,21,22^.

However, limited definitive data exists for PPM in the setting of TAVR, and interest in this area is intensifying. The pooled incidence of PPM following TAVR was 33%, while the combined prevalence of moderate and severe PPM was 25% and 11% respectively in our meta-analysis. The definition of PPM in our eligible studies was based on measured EOA indexed to BSA. However, regarding the relationship between left ventricular output tract diameter (LVOTd) and EOA, the accuracy measurements of LVOTd is vital for the reporting prevalence of PPM. In our study, the incidence of PPM was higher in the method using proximal to leaflet, in comparison with that using underneath stent (proximal to leaflet 46.0% vs underneath stent 36.0%, P = 0.002).While, Clavel *et al*.^42^ revealed that LVOTd being measured underneath the stent was more stable and reduplicated than that proximal to prosthesis cusps. And it was the way underneath the stent that correlated better with transthoracic gradient^42^. Therefore, the definition of PPM according to LVOTd being measured underneath the stent may be more accurate than that being measured proximal to prosthesis cusps.

In our study, we found that MCV had lower incidence of overall and moderate PPM than ESV. Consistently, the pooled results showed that MCV^9,12–14,17,18,22,31,33,37^ had lower mean trans-vavular mean gradient than ESV^9–11,14,16,19–21,23,25,32^ (MCV, 9.3 mmHg vs ESV, 10.2 mmHg, P < 0.001). Additionally, the randomized CHOICE trial^43^ also revealed that MCV was associated with lower trans-valvular mean gradient than ESV (MCV vs ESV, 8 mmHg vs 9 mmHg, p = 0.004), albeit no reported data on the comparison of PPM. This difference may be attributed to the different design. As we all known, MCV is a supra-annular design with an hour-glass shape stent and a flared inflow. A self-expanding design may confer greater conformity to the native valve and left ventricular outflow tract morphology than the intra-annular design of ESV^14^. Therefore, when patients with high risk for PPM, a self-expandable MCV may be a preferential choice.

Interestingly, PPM was not associated with increased short-and mid-term all-cause mortality following TAVR in the present meta-analysis, which was in line with that from the Placement of AoRTic TraNscathetER Valves (PARTNER) trial^11^. Overall, moderate and severe PPM did not show a detrimental effect on short- and mid-term survival in our meta-analysis; a relationship was shown, however, in some studies, with severe PPM predicting mid-term mortality in a multivariable analysis^15,32^ and in a subgroup analysis of the PARTNER trial that excluded patients with aortic regurgitation^11^. This is in contrast to established data from the surgical literature, with moderate and severe PPM following SAVR shown to have a higher mortality in pooled data from 34 and 58 studies recruiting 27,186 and 40,381 patients respectively^5,6^. This paradox may be related to the influence of individual preoperative characteristics and baseline comorbidities^38,40^. Patients undergoing TAVR thus far have been older than that of SAVR, potentially with lower basic activity requirements, and multiple comorbidities that compete with the influence of PPM. Furthermore, Price *et al*.^38^ and Dayan *et al*.^5^ reported that patients older than 70 years with PPM following SAVR were not susceptible to impaired survival and congestive heart failure regardless of LV function and LV mass regression. Additionally, compared with SAVR, TAVR was demonstrated to have better hemodynamic characteristics resulting in less impairment of coronary flow reserve which may be another key factor to explain the difference^11,21,22^. Previous studies also reported that extracorporeal circulation during SAVR was associated with increased risk for systemic inflammatory response syndrome and myocardial infarction, which would impair perioperative survival and impede LV function recovery^44,45^. This would also explain the different prognosis effect of PPM between SAVR and TAVR. Nonetheless, patients with PPM has been shown to impede the improvement of LV mass regression and NYHA class after TAVR, but that the extent of such an improvement varies widely between individuals post procedure^11,12^. Therefore, with the extension of TAVR to younger patients, the detrimental effect of PPM following TAVR could conceivably become apparent.

In our study, we found that patients with more severely stenotic aortic valves were more likely to encounter PPM. When the transcatheter valve was implanted in a severely stenotic native aortic valve especially that with heavy leaflet calcification, incomplete expansion of the stent frame may occur and result in PPM^46^. Under such circumstances, post-dilation, an additional procedure could be considered to reduce the risk of PPM, especially in the presence of substantial paravalvular leak^46^. Previous study reported there was no significant difference on the incidence of post-dilation between patients with prior balloon valvulopasty and without prior balloon valvuloplasty, thus whether prior balloon valvuloplasty before TAVR would influence the occurrence rate of PPM should be evaluated in further studies^47^. Moreover, because there are limited sizes of prostheses available for a wide range of annular sizes, patients with larger BSA were susceptible to a higher occurrence rate of PPM which is in lines with that in SAVR^48^. The present meta-analysis found there was significant correlation between annulus diameter and the prevalence of PPM, which was consistent with PARTNER trail^39^. Therefore, an optimized method for measuring annular size was a crucial approach to reducing the occurrence of PPM^39^. Regarding the method of sizing the annulus diameters before TAVR, 3D echocardiography and 3D multidetector CT were superior to 2D echocardiography that could decrease the incidence of PPM^19,37^. In addition, Jilaihawi *et al*.^17^ demonstrated that optimal position of the implanted valve (defined in this early study as 5–10 mm below the native non-coronary cusp) was associated with a lower incidence of PPM with the MCV device. Based on this potential relationship, we should strive to obtain an optimal position by selecting suitable projection direction and careful deployment.

### Limitation

There were several limitations in the present systematic review and meta-analysis. Firstly, although significant heterogeneity was noted, subgroup analysis including study design, patients’ recruitment time and time to define PPM was conducted to reveal the source of heterogeneity. While, the result of sensitivity analysis confirmed the robustness of our pooled estimate. Secondly, cumulative survival data was sparse and we relied on binary survival data to generate ORs rather than Hazard Ratios (HR). Although the combined ORs referring to mortality did not exclude the influence of potential mediators, the unadjusted pooled ORs can be elucidated as the entire causal effects of PPM, which might be more clinically relevant. Thirdly, the TAVR series had relatively short follow-up compared to SAVR series, so the results comparing the incidence of PPM between TAVR and SAVR should be validated by further studies. Finally, the preventive measures of PPM presented here were based on isolated studies and would therefore require further validation in future large-scale prospective studies.

## Conclusion

In the presented TAVR was associated with a significantly lower risk of overall, moderate and severe PPM compared with SAVR. Reported frequencies of overall and moderate PPM following TAVR-MCV was lower than that of TAVR-ESV. Although PPM after TAVR did not display a detrimental effect on short- and mid-term outcomes, regardless of the severity of PPM, the impact on long-term outcomes, particularly relevant to the increasingly younger TAVR population, remains to be elucidated. Until such data is available, it seems reasonable to strive to optimize TAVR hemodynamic performance and reduce the occurrence of PPM.

## Methods

### Registration

Our systematic review was registered online in PROSPERO (registration number: CRD42014015518).

### Data sources and study selection

The PubMed online database (between January 1, 2002 and Oct 19, 2016) was searched by using the following search strategy: (prosthesis patient mismatch OR patient prosthesis mismatch) AND (percutaneous OR transcatheter OR transfemoral OR transapical OR transartery) AND (aortic valve) AND (replacement OR implantation). Besides, reference lists of pertinent articles were also screened manually for potentially relevant citations. All citations were initially screened at the level of title and abstract. And then full-length articles were retrieved for further evaluation. Two authors screened citations separately. If controversies existed, a discussion was made to achieve consensus.

### Study inclusion criteria

Articles were included if they 1) reported the specific number or incidence of PPM; 2) presented the predictive factors or preventive measures of PPM; 3) displayed the particular number of death or survival curve of PPM; 4) were human studies and published in English. The exclusion criteria were abstract, editorials, reviews and case reports. The process of study selection was illustrated in Fig. 1.

### Data extraction and quality assessment

Two authors (YBL and YJL) independently extracted the specific characteristics from eligible studies including author, PPM definition, baseline profiles and mortality. If mortality or number of death was not presented directly, we used digitizing software (Engauge Digtizer 4.1) to gain data from survival cure^49^. PPM in the present meta-analysis was defined if indexed EOA was less than 0.85 cm^2^/m^2^, moderate PPM defined as ≥0.65 cm^2^/m^2^ and ≤0.85 cm^2^/m^2^, and severe PPM defined as <0.65 cm^2^/m^2^. A table was designed to record the data. Study quality was assessed using the Newcastle-Ottawa Quality Assessment Scale (NOS)^50^.

### Data synthesis and analysis

Pooled incidences and odds ratios (OR) were acquired using the Comprehensive Meta-Analysis Software Version 2. Heterogeneity was assessed by calculating the I^2^ statistic and its P value. If the I^2^ statistic was more than 50% and its P value was less than 0.05, a random-effects model was used to obtain the combined effect estimates. Two-sided P values of 0.05 were considered statistically significant.

### Publications bias analysis

Publication bias was evaluated by visual inspection of the symmetry of the funnel plot and by Egger’s test. Two-sided P values of 0.05 in the Egger’s test were not associated with significant publication bias. Besides, if the number of pooled studies was small, publication bias was not performed.

### Sensitivity and subgroup analysis

We performed sensitivity analysis by discarding one study at a time and repeating the meta-analysis to examine the robustness of the pooled results. Sensitivity analysis was not conducted unless there were sufficient studies to gain the pooled estimate. Additionally, subgroup analysis was carried out according to the valve type, study design, mean Logistic EuroSCORE, patients’ recruitment time and the time point where PPM was analyzed to explore the source of heterogeneity.

Present systematic review and meta-analysis was conducted and reported according to the recommendations of the Meta-analysis Of Observational Studies in Epidemiology (MOOSE) group^51^.

### Data availability statement

The datasets generated during and/or analysed during the current study are available from the corresponding author on reasonable request.

## Electronic supplementary material

Supplementary table
