# Supplementary material for: Incidence, Predictors and Outcome of Prosthesis-Patient Mismatch after Transcatheter Aortic Valve Replacement: a Systematic Review and Meta-analysis
Source: Sci Rep. 2017 Nov 8;7:15014. doi: 10.1038/s41598-017-15396-4 (PMC5678180; doi:10.1038/s41598-017-15396-4)
Supplement: Supplementary file 1 — Supplementary table [file 41598_2017_15396_MOESM1_ESM.doc]

**Incidence, Predictors and Outcome of Prosthesis-Patient Mismatch after Transcatheter Aortic Valve Replacement: a Systematic Review and Meta-analysis**

Yan-biao Liao^1*^, MD, Yi-jian Li^1*^, MD, Jun-li, Li^1^, MD, Zhen-gang Zhao^1^, MD, Xin Wei^1^, MD, Jiay-yu Tsauo^1^, MS, Tian-yuan Xiong^1^, MD, Yuan-ning Xu^1^, MD, Yuan Feng^1^**^†^**, MD, Mao Chen^1^**^†^**, MD, PhD

**Supplemental Table 1 characteristic of overall** included studies

| Author | N | Recruitment time | Age (yrs) | EuroSCORE (%) | Aortic annulus(mm) | Valve size (mm) | Study design | Methodology  (measure LVOTd) | Mean gradient  (Post-TAVR) | Follow up | Time to define PPM | Incidence |
| --- | --- | --- | --- | --- | --- | --- | --- | --- | --- | --- | --- | --- |
| Tzikas^15^ 2010 | 74 | ng | 81±7 | 15±8 | ng | MCV: 26 (50%)  MCV: 29 (50%) | retrospective | underneath the prosthesis stent | MCV: 9.0±5.0 | 6 months | discharge | Severe:16% Moderate:23% |
| Sherif^16^ 2010^*^ | 56 | 2007-2009 | 80.5±7.5 | 22.6±11.3 | TEE: 22.3±1.8 | MCV: 26 (55%)  MCV: 29 (45%) | prospective | ng | MCV: 10.3±4.4 | 1 month | 1 month | Severe: 0 Moderate:17.8% |
| Pibarot^11^ 2014(RCT) | 304 | 2007-2009 | 84.1±7.1 | 26.0±5.7 | TEE: 20.1±2.4 | ESV | RCT | underneath the prosthesis stent | ESV**^†^**: 10.8±4.6 | 2 years | 7 to 180 days | Severe:19.7% Moderate:26.6% |
| Pibarot^11^ 2014(NRCT) | 1637 | 2009-2011 | 85.1±6.5 | 23.0±5.1 | TEE: 18.9±2.7 | ESV | prospective | underneath the prosthesis stent | ESV**^†^**: 10.3±4.4 | 2 years | 7 to 180 day | Severe:13.6% Moderate:30.2% |
| Franco^17^ 2013 | 82 | ng | 83±6 | ng | TEE: 20.5±1.1 | MCV: 26 (50%)  ESV: 26 (50%) | prospective | underneath the prosthesis stent | MCV: 7.9±3.1  ESV: 9.7±3.8 | 1 year | discharge | Severe:9.8% Moderate:35.4% |
| Author | N | Recruitment time | Age (yrs) | EuroSCORE (%) | Aortic annulus(mm) | Valve size | Study design | Methodology  (measure LVOTd) | Mean gradient  (Post-TAVR) | Follow up | Time to define PPM | Incidence |
| Garcia^18^ 2013 | 166 | 2008-2011 | 79.3±6.2 | 19.6±11.8 | 21.9±1.7**^‡^** | MCV: 26 (55%)  MCV: 29 (44%) | prospective | underneath the prosthesis stent | MCV: ng | 2 years | 3 days | Severe:9% Moderate:34.9% |
| Kalavrouziotis^19^ 2011 | 35 | 2007-2010 | 79.2±9.4 | 18.8±14.1 | TEE: 18.5±0.9 | ESV: 23 (100%) | prospective | underneath the prosthesis stent | ESV: 11.7±4.8 | 1 year | pre-discharge | Severe:5.9% Moderate:32.4% |
| Jilaihawi^20^ 2011 | 50 | 2007-2008 | 83±6 | 22±13 | TEE: 23.1±1.8 | MCV: 26 (60%)  MCV: 29 (40%) | retrospective | underneath the prosthesis leaflets | MCV: 8.1±3.3 | ng | pre-discharge | Overall:32% |
| Gotzmann^21^ 2010 | 39 | 2008-2009 | 78.5±6.8 | 17.7±14.7 | ng | ng | prospective | underneath the prosthesis stent | MCV: 10.2±4 | 6 months | pre-discharge | Severe:2.6% Moderate:7.8% |
| Freeman^22^ 2013 | 128 | 2009-2012 | 82.1±7.6 | ng | TEE: 22.6±2.1 | ESV: 23 (47%)  ESV: 26 (45%) | prospective | underneath the prosthesis stent | ESV: 11.6±5.1 | ng | pre-discharge | Severe:9.4% Moderate:42.2% |
| Ewe^23^ 2011 | 165 | ng | 80.5±7.1 | 22±11.9 | TEE:21.8±2.2 | ESV: 23 (38%)  ESV: 26 (62%) | prospective | underneath the prosthesis stent | ESV: 11.5±4.1 | 34 months | pre-discharge | Overall:18.2% |
| Author | N | Recruitment time | Age (yrs) | EuroSCORE (%) | Aortic annulus(mm) | Valve size | Study design | Methodology  (measure LVOTd) | Mean gradient  (Post-TAVR) | Follow up | Time to define PPM | Incidence |
| Silva^40^ 2014 | 45 | 2008-2011 | 84.6±5.5 | ng | TTE:20.8±2.3  TEE: 23.0 ±2.0 | MCV: 26 (53%)  MCV: 29 (47%) | prospective | underneath the prosthesis leaflets | MCV: 9.4±4.0 | 3 months | pre-discharge | Severe:15.6% Moderate:26.7% |
| Clavel^24^ 2010 | 83 | 2005-2009 | 81±8 | 32±18 | ng | ESV: 23 (48%)  ESV: 26 (52%) | retrospective | ng | ESV: 10±5 | 1 year | 1 year | Severe:16% |
| Bleiziffer^9^ 2013 | 149 | 2007-2009 | 81±6 | 20±13 | TEE: 23.0±2.0 | MCV26, ESV23 MCV29, ESV26 | prospective | 1cm below the prosthesis leaflets | MCV: 11.1±4.2  ESV: 12.0±4.0 | 6 months | 6 months | Severe:14.8% Moderate:46.3% |
| Kukucka^10^ 2013 | 272 | 2008-2011 | 80±8 | 37.8±20.2 | TEE: 21.8±1.6 | ESV: 23 (33%)  ESV: 26 (67%) | retrospective | underneath the prosthesis stent | ESV: 5.4±3.0 | 28 months | pre-discharge | Severe:7.6% Moderate:27.3% |
| Linden^26^ 2013 | 112 | 2006-2009 | 82.4±5.6 | 30.4±14.8 | TTE: 22.7±1.4 | ESV: 23(26%)  ESV: 26(74%) | retrospective | underneath the prosthesis stent | ESV: 8.4±3.8 | 1 year | pre-discharge | Severe:9.8% Moderate:28.6% |
| Giannini^25^ 2011 | 48 | ng | 82.2±5.4 | 22.3±12.4 | TTE: 21.7±2.1 | MCV: 26(65%)  MCV: 29(35%) | retrospective | underneath the prosthesis stent | MCV: 10.4±4.8 | 1 year | discharge | Severe:9% |
| Author | N | Recruitment time | Age (yrs) | EuroSCORE (%) | Aortic annulus(mm) | Valve size | Study design | Methodology  (measure LVOTd) | Mean gradient  (Post-TAVR) | Follow up | Time to define PPM | Incidence |
| Seiffert^27^2012^§^ | 11 | 2008-2011 | 79.1±6.3 | ng | ng | ESV: 23(91%)  ESV: 26(9%) | prospective | underneath the prosthesis stent | ESV: 17.9±8.3 | 6 months | discharge | Severe:45.4%  Moderate:45.5% |
| Zorn^36^ 2016 | 367 | 2011-2012 | 82.9±7.1 | 17.2±12.6 | 22.2±2.0**^‡^** | MCV | RCT | underneath the prosthesis stent | MCV: 9.1±3.5 | 1 year | discharge | Severe:7.0% Moderate:19.2% |
| Utsunomiya^35^ 2016 | 131 | 2013-2014 | 83±8 | ng | ng | ESV: 23(38%) ESV: 26(44%) | retrospective | underneath the prosthesis stent | ESV: 10.0±4.0 | 2.4 years | Discharge | Severe: 12% Moderate: 32% |
| Thyregod^34^ 2016 | 121 | 2009-2012 | 79.1±4.9 | 8.4±4.1 | TEE: 22.5±2.0 | MCV: 26(41%) MCV: 29(49%) | RCT | ng | MCV: 8.3±4.0 | 2 years | 3 month | Severe: 14.0% Moderate: 21.5% |
| Poulin^31^ 2016 | 102 | 2007-2013 | 83±5.6 | 19±13 | TTE: 21.1±2.2 | MCV, ESV | retrospective | underneath the prosthesis stent | 10.8±4.5 | 1 year | 2-12 months | Severe: 9% Moderate: 21% |
| Del Trigo^28^ 2016 | 62 | ng | 81.5±6.2 | ng | TTE: 19.6±1.3 | PV: 22(35.4%) ESV: 40(64.6%) | retrospective | underneath the prosthesis stent | PV: 9.8±1.1 ESV: 10.4±3.7 | 1 month | 1 month | Severe:12.3% Moderate: 14.0% |
| Author | N | Recruitment time | Age (yrs) | EuroSCORE (%) | Aortic annulus(mm) | Valve size | Study design | Methodology  (measure LVOTd) | Mean gradient  (Post-TAVR) | Follow up | Time to define PPM | Incidence |
| Spangenberg^32^ 2015 | 95 | 2011-2013 | 82.4±6.5 | 21.7±18.3 | ng | MCV, ESV, PV | prospective | ng | 6.4±4.0 | 1 year | Pre-discharge | Severe: 0 Moderate: 4.2% |
| Laflamme^30^ 2015 | 122 | 2012-2013 | 79.0±7.7 | ng | ng | MCV, ESV | retrospective | ng | 9.4±3.6 | 1 year | Discharge | Severe:21.3% Moderate: 32.0% |
| Kamperidis^29^ 2015 | 40 | 2007-2013 | 79±5.9 | 15.5±8.4 | CT: 24.0±2.0 | MCV, ESV | retrospective | ng | 8.14±4.21 | 2 years | discharge | Severe: 10.3% Moderate: 20.5% |
| Subban^33^ 2014^§^ | 12 | 2009-2014 | 78.5±7.0 | ng | ng | MCV: 26(25%) ESV: 4(33.3%) | retrospective | ng | 15±8 | 2 years | ng | Overall: 33.3% |
| Unbehaun^38^ 2013 | 78 | 2011-2012 | 78±3.1 | 26±15.3 | TEE: 25.3±1.0 | ESV: 26(51.3%) ESV: 29(48.7%) | retrospective | ng | ng | 1 year | 1 month | Severe: 0 Moderate: 6.3% |
| Finkelstein^37^ 2014 | 86 | 2009-2011 | 82.4±5.05 | ng | ng | ESV, MCV | retrospective | ng | 9.08±3.62 | 1.6 years | pre-discharge | Severe: 6% Moderate: 17% |
| Author | N | Recruitment time | Age (yrs) | EuroSCORE (%) | Aortic annulus(mm) | Valve size | Study design | Methodology  (measure LVOTd) | Mean gradient  (Post-TAVR) | Follow up | Time to define PPM | Incidence |
| Schmidt^39^ 2016**^§^** | 19 | 2008-2015 | 77.8±6.6 | 31.6±20.1 | ng | MCV, ESV | retrospective | ng | 7.5±4.5 | 12 months | ng | Severe: 0 Moderate:11% |

* indicate the definition of PPM (moderate PPM: 0.6 cm^2^/m^2^<EOA<0.9 cm^2^/m^2^, severe PPM: EOA<0.6 cm^2^/m^2^) was not consistent with other studies (moderate PPM: 0.65 cm^2^/m^2^<EOA<0.85 cm^2^/m^2^, severe PPM: EOA<0.65 cm^2^/m^2^); † indicates the number of patients available for mean gradient were 279 and 1359 separately; **^‡^** indicates the method for valve sizing is not given; ng not given; CT computed tomography; **^§^** indicates the study reported valve-in-valve procedure; LVOTd left ventricular output tract diameter; TAVR transcatheter aortic valve replacement; RCT randomized clinical trial; NRCT indicates non-randomized clinical trial.

**Supplemental Table 2 Pooled incidence of Prosthesis-Patient Mismatch after transcatheter aortic valve replacement**

| Subgroup | | No. of studies | No. of patients | Incidences | I^2^ for heterogeneity | Model | P for Egger’s |
| --- | --- | --- | --- | --- | --- | --- | --- |
| Overall |  | 25^9-11, 15-23, 26, 28-34, 36-38, 40^ | 4,399 | 0.33 (0.29-0.38) | 88.8 | Random | 0.1 |
| Moderate | | 23^9-11, 15-19, 21, 22, 26, 28-32, 34-38, 40^ | 4,303 | 0.25 (0.22-0.29) | 81.7 | Random | 0.3 |
| Severe | | 25^9-11, 15-19, 21, 22, 25, 26, 28-32, 34-38, 40^ | 4,434 | 0.11 (0.09-0.13) | 65.7 | Random | 0.05 |
| LVOTd measurement method | |  |  |  |  |  |  |
| PPM (underneath stent) | | 15^10, 11, 15, 17-19, 21-23, 26, 28, 31, 35, 36^ | 3,373 | 0.36 (0.31-0.41) | 86.4 | Random | 0.10 |
| PPM (proximal to leaflets) | | 3^9, 20, 40^ | 244 | 0.46 (0.29-0.65) | 85.5 | Random | NA |
| Valve type | |  |  |  |  |  |  |
| PPM-MCV | | 8^15-18, 21, 34, 36, 40^ | 909 | 0.32 (0.25-0.40) | 78.6 | Random | 0.72 |
| Moderate PPM-MCV | | 8^15-18, 21, 34, 36, 40^ | 909 | 0.23 (0.18-0.28) | 67.7 | Random | 0.51 |
| Severe PPM-MCV | | 9^15-18, 21, 25, 34, 36, 40^ | 957 | 0.10 (0.08-0.12) | 48.7 | Fixed | 0.51 |
| PPM-ESV | | 11^9-11, 17, 19, 22, 23, 26, 35, 38^ | 3,052 | 0.40 (0.33-0.47) | 90.0 | Random | 0.42 |
| Moderate PPM-ESV | | 10^9-11, 17, 19, 22, 26, 35, 38^ | 2,887 | 0.32 (0.27-0.37) | 81.7 | Random | 0.87 |
| Severe PPM-ESV | | 11^9-11, 17, 19, 22, 24, 26, 35, 38^ | 3,036 | 0.12 (0.10-0.15) | 65.7 | Random | 0.10 |
| Recruitment time | |  |  |  |  |  |  |
| PPM (early recruitment) | | 5^11, 19, 26, 29, 31^ | 568 | 0.38 (0.31-0.45) | 57.7 | Random | 0.13 |
| PPM (later recruitment) | | 13^10, 11, 18, 21, 22, 30, 32-38^ | 2,976 | 0.31 (0.30-0.40) | 90.9 | Random | 0.05 |
| Moderate PPM (early recruitment) | | 5^11, 19, 26, 29, 31^ | 568 | 0.26 (0.23-0.30) | 0 | Fixed | 0.78 |
| Moderate PPM (later recruitment) | | 12^10, 11, 18, 21, 22, 30, 32, 34-38^ | 2,964 | 0.23 (0.19-0.29) | 86.5 | Random | 0.06 |
| Severe PPM (early recruitment) | | 5^11, 19, 26, 29, 31^ | 568 | 0.15 (0.12-0.19) | 68.4 | Random | 0.05 |
| Severe PPM (later recruitment) | | 12^10, 11, 18, 21, 22, 30, 32, 34-38^ | 2964 | 0.10 (0.07-0.13) | 75.1 | Random | 0.02 |
| Risk score | |  |  |  |  |  |  |
| PPM (low risk) | | 8^15, 18, 19, 21, 29, 31, 34, 36^ | 944 | 0.33 (0.26-0.40) | 73.3 | Random | 0.87 |
| PPM (high risk) | | 9^9-11, 20, 23, 26, 32, 38^ | 2,559 | 0.30 (0.23-0.40) | 93.5 | Random | 0.08 |
| Moderate PPM (low risk) | | 8^15, 18, 19, 21, 29, 31, 34, 36^ | 944 | 0.23 (0.18-0.29) | 68.5 | Random | 0.65 |
| Subgroup | | No. of studies | No. of patients | Incidences | I^2^ for heterogeneity | Model | P for Egger’s |
| Moderate PPM (high risk) | | 8^9-11, 16, 26, 32, 38^ | 2,400 | 0.24 (0.18-0.31) | 88.3 | Random | 0.15 |
| Severe PPM (low risk) | | 8^11, 15, 18, 19, 21, 31, 34, 36^ | 944 | 0.10 (0.08-0.12) | 38.8 | Fixed | 0.67 |
| Severe PPM (high risk) | | 9^9-11, 24-26, 32, 38^ | 2,475 | 0.12 (0.09-0.16) | 78.8 | Random | 0.12 |
| Study design | |  |  |  |  |  |  |
| PPM (prospective design) | | 10^9, 11, 17-19, 21-23, 32, 40^ | 2,541 | 0.35 (0.27-0.44) | 91.4 | Random | 0.24 |
| PPM (retrospective design) | | 11^10, 15, 20, 26, 28-31, 35, 37, 38^ | 1,129 | 0.33 (0.27-0.40) | 80.3 | Random | 0.06 |
| PPM (RCT) | | 3^11, 34, 36^ | 792 | 0.36 (0.24-0.50) | 93.1 | Random | 0.95 |
| Moderate PPM (prospective design) | | 10^9, 11, 16-19, 21, 22, 32, 40^ | 2,432 | 0.28 (0.22-0.36) | 84.6 | Random | 0.53 |
| Moderate PPM (retrospective design) | | 10^10, 15, 26, 28-31, 35, 37, 38^ | 1,079 | 0.23 (0.19-0.28) | 67.3 | Random | 0.05 |
| Moderate PPM (RCT) | | 3^11, 34, 36^ | 792 | 0.22 (0.18-0.28) | 62.0 | Random | 0.89 |
| Severe PPM (prospective design) | | 9^9, 11, 17-19, 21, 22, 32, 40^ | 2,376 | 0.11 (0.09-0.14) | 48.9 | Fixed | 0.1 |
| Severe PPM (retrospective design) | | 12^10, 15, 24-26, 28-31, 35, 37, 38^ | 1,210 | 0.11 (0.09-0.13) | 58.0 | Random | 0.08 |
| Severe PPM (RCT) | | 3^11, 34, 36^ | 792 | 0.13 (0.06-0.24) | 91.0 | Random | 0.60 |
| Time to define PPM | |  |  |  |  |  |  |
| PPM (pre-discharge) | | 11^10, 18-23, 26, 32, 37, 40^ | 1,193 | 0.30 (0.22-0.38) | 87.4 | Random | 0.09 |
| PPM (discharge) | | 6^15, 17, 29, 30, 35, 36^ | 816 | 0.40 (0.30-0.50) | 86.6 | Random | 0.35 |
| Moderate PPM (pre-discharge) | | 9^10, 18, 19, 21, 22, 26, 32, 37, 40^ | 978 | 0.24 (0.18-0.32) | 82.5 | Random | 0.06 |
| Moderate PPM (discharge) | | 6^15, 17, 29, 30, 35, 36^ | 816 | 0.27 (0.21-0.34) | 72.3 | Random | 0.54 |
| Severe PPM (pre-discharge) | | 9^10, 18, 19, 21, 22, 26, 32, 37, 40^ | 978 | 0.09 (0.07-0.11) | 21.2 | Fixed | 0.09 |
| Severe PPM (discharge) | | 7^15, 17, 25, 29, 30, 35, 36^ | 864 | 0.12 (0.10-0.14) | 70.1 | Random | 0.9 |

Abbreviations: PPM Prosthesis-Patient Mismatch; MCV Medtronic CoreValve; ESV Edwards Sapien Valve; OR Odds Ratio; TAVR transcatheter aortic valve replacement; SAVR surgical aortic valve replacement; NA not applicable; LVOTd left ventricular output tract diameter; high risk indicates that Logistic EuroSCORE was more than 20%, low risk indicates Logistic EuroSCORE was less than 20%.
